# New Insights into [FeFe] Hydrogenase Activation and Maturase Function

**DOI:** 10.1371/journal.pone.0045850

**Published:** 2012-09-25

**Authors:** Jon M. Kuchenreuther, R. David Britt, James R. Swartz

**Affiliations:** 1 Department of Chemical Engineering, Stanford University, Stanford, California, United States of America; 2 Department of Chemistry, University of California Davis, Davis, California, United States of America; 3 Department of Bioengineering, Stanford University, Stanford, California, United States of America; National Institute for Medical Research, Medical Research Council, London, United Kingdom

## Abstract

[FeFe] hydrogenases catalyze H_2_ production using the H-cluster, an iron-sulfur cofactor that contains carbon monoxide (CO), cyanide (CN^–^), and a dithiolate bridging ligand. The HydE, HydF, and HydG maturases assist in assembling the H-cluster and maturing hydrogenases into their catalytically active form. Characterization of these maturases and *in vitro* hydrogenase activation methods have helped elucidate steps in the H-cluster biosynthetic pathway such as the HydG-catalyzed generation of the CO and CN^–^ ligands from free tyrosine. We have refined our cell-free approach for H-cluster synthesis and hydrogenase maturation by using separately expressed and purified HydE, HydF, and HydG. In this report, we illustrate how substrates and protein constituents influence hydrogenase activation, and for the first time, we show that each maturase can function catalytically during the maturation process. With precise control over the biomolecular components, we also provide evidence for H-cluster synthesis in the absence of either HydE or HydF, and we further show that hydrogenase activation can occur without exogenous tyrosine. Given these findings, we suggest a new reaction sequence for the [FeFe] hydrogenase maturation pathway. In our model, HydG independently synthesizes an iron-based compound with CO and CN^–^ ligands that is a precursor to the H-cluster [2Fe]_H_ subunit, and which we have termed HydG-co. We further propose that HydF is a transferase that stabilizes HydG-co and also shuttles the complete [2Fe]_H_ subcluster to the hydrogenase, a translocation process that may be catalyzed by HydE. In summary, this report describes the first example of reconstructing the [FeFe] hydrogenase maturation pathway using purified maturases and subsequently utilizing this *in vitro* system to better understand the roles of HydE, HydF, and HydG.

## Introduction

Metalloenzymes such as hydrogenases and nitrogenases harbor complex metal clusters that catalyze industrially relevant reactions. Hydrogenase cofactors such as the [FeFe]-based H-cluster catalyze the redox interconversion of protons and H_2_. The [FeFe] hydrogenase H-cluster consists of a [4Fe–4S] cubane cluster (the [4Fe–4S]_H_ subcluster) that is connected by a cysteinyl sulfur ligand to a catalytic two-iron unit (the [2Fe]_H_ subcluster) that coordinates multiple nonprotein ligands: three CO, two CN^–^, and a dithiol bridge (DTMX) that is arguably dithiomethylamine or dithiomethylether [1], [2], [3], [4]. Several intriguing applications have stimulated research to develop technologies that use hydrogenases or synthetic catalysts inspired by them. A more thorough understanding of H-cluster synthesis would likely facilitate the engineering of such biotechnologies, and recent studies have provided valuable insights toward fully elucidating the [FeFe] hydrogenase biosynthetic pathway.

HydE, HydF, and HydG – termed the Hyd maturases – are the three [FeS] cluster accessory proteins that directly participate in the assembly of the [2Fe]_H_ subcluster and the maturation of [FeFe] hydrogenases [5]. HydG is a radical *S*-adenosyl methionine (SAM) enzyme that catalyzes the formation of CO and CN^–^ from free tyrosine [6], [7], [8], to provide the three CO and two CN^–^ [2Fe]_H_ subcluster ligands [9]. HydE is also a radical SAM protein [10], yet no substrates besides SAM and no precise role of this maturase have been identified. In addition, both the origin of the DTMX ligand and the protein that makes it have not been determined, although HydE [11], [12] and HydG [13] were separately hypothesized as the maturase responsible for synthesizing this dithiol bridge. The HydF maturase is a GTPase, yet the purpose of GTP hydrolysis is presently unresolved [12], [14]. When co-expressed with HydE and HydG, HydF coordinates an iron cluster with CO and CN^–^ adducts [12], [15] and is also able to activate the [FeFe] hydrogenase *in vitro* [16]. These results, together with EPR spectroscopy of HydF expressed in a genetic background devoid of HydE and HydG, have led to the theory that the HydF maturase harbors a [2Fe–2S] cluster that serves as the scaffold for the [2Fe]_H_ subcluster, onto which CO and CN^–^ molecules generated by HydG along with the DTMX ligand are assembled [12]. The activation of the hydrogenase apoprotein has been suggested to be a two-step process, where the [4Fe–4S]_H_ subcluster is first assembled, followed by [2Fe]_H_ subcluster transfer from HydF to the hydrogenase, producing the active enzyme [17].

*In vitro* protein biochemistry studies are a valuable means to identify cofactors and substrates as well as to characterize enzyme functionality. While investigating a single protein can be straightforward, reconstituting a multi-component reaction sequence requires an understanding of the necessary proteins and the influential small molecular weight substrates. *In vitro* assembly protocols that use a minimal set of purified enzymes have been developed for complex metal clusters such as the nitrogenase iron-molybdenum cofactor (FeMo-co), and subsequent experimentation has then provided insights to further define the activation sequence [18], [19]. Approaches for *in vitro* [FeFe] hydrogenase maturation have been established that utilize purified HydF with a bound [2Fe]_H_ subcluster, although 100-fold excess HydF was required to mature roughly 15% of the hydrogenase [16]. To date, we have reported the only examples of *in vitro* systems in which exogenous substrates have enhanced hydrogenase maturation, 100% of the hydrogenase has been activated, and cell-free synthesis of the [2Fe]_H_ subcluster has been demonstrated [9], [20]. Nevertheless, no *in vitro* approach that uses the maturases in their pure forms has so far been detailed.

In this work, we describe the first *in vitro* system for activating [FeFe] hydrogenases using individually expressed and purified HydE, HydF, and HydG. Reconstructing H-cluster biosynthesis in a cell-free environment with purified maturases allows for precise control over the presence and the concentrations of these biomolecular constituents. We detail how excluding small molecule substrates, an *Escherichia coli* lysate from the strain BL21(DE3) Δ*iscR*, and each maturase impact activation of the CpI hydrogenase, the HydA enzyme from *Clostridium pasteurianum*. We also explore how maturase concentrations affect CpI activation rates, providing the first evidence that HydE, HydF, and HydG each function catalytically during the maturation reaction. Together, our results lend new insights regarding the roles of the three Hyd maturases, from which we propose a new model for H-cluster synthesis and [FeFe] hydrogenase maturation.

## Results and Discussion

### Influence of Exogenous Small Molecules on [FeFe] Hydrogenase Maturation

Upon modifying our first *in vitro* hydrogenase activation system [20] to incorporate separately and anaerobically expressed and purified maturases, we observed that tyrosine (L-Tyr), SAM, GTP, pyridoxal-5′-phosphate (PLP), cysteine (L-Cys), ferrous ammonium sulfate (Fe^2+^), sodium sulfide (S^2–^), dithiothreitol (DTT), and sodium dithionite (DTH) each had beneficial effects. The addition of all of these substrates resulted in complete activation, but either lower or no hydrogenase activities were seen when certain molecules were not included (Table 1). For example, excluding tyrosine or PLP led to partial activation, and no CpI activity was detected when either SAM or GTP was excluded from the reaction mixtures. While individually excluding Fe^2+^, S^2–^, DTT, DTH, or cysteine had no observable effects, conditions were identified in which CpI was not activated when certain combinations of these five chemicals were excluded (e.g. Δ(DTT, DTH, Fe, S)). For these experiments, the *E. coli* lysates from the Δ*iscR* strain were buffer exchanged with desalting columns to significantly reduce the probability that adventitious small molecular weight substrates were influencing hydrogenase maturation.

**Table 1:** Effects of exogenous substrates on *in vitro* hydrogenase activation.

Small Molecules Added	Excluded	Activity†
DTT, DTH, Fe, S, L-Cys, L-Tyr, GTP, PLP, SAM	None (positive control)	633±64
DTH, Fe, S, L-Cys, L-Tyr, GTP, PLP, SAM	ΔDTT	707±48
DTT, Fe, S, L-Cys, L-Tyr, GTP, PLP, SAM	ΔDTH	733±109
Fe, S, L-Cys, L-Tyr, GTP, PLP, SAM	Δ(DTT, DTH)	713±46
DTT, DTH, S, L-Cys, L-Tyr, GTP, PLP, SAM	ΔFe	710±72
DTT, DTH, Fe, L-Cys, L-Tyr, GTP, PLP, SAM	ΔS	698±64
DTH, L-Cys, L-Tyr, GTP, PLP, SAM	Δ(DTT, Fe, S)	494±49
DTT, L-Cys, L-Tyr, GTP, PLP, SAM	Δ(DTH, Fe, S)	684±9
L-Cys, L-Tyr, GTP, PLP, SAM	Δ(DTT, DTH, Fe, S)	0
DTT, DTH, Fe, S, L-Tyr, GTP, PLP, SAM	ΔL-Cys	677±29
DTT, DTH, Fe, S, L-Tyr, GTP, PLP, SAM, **D-Cys**	ΔL-Cys	593±105
DTH, L-Tyr, GTP, PLP, SAM	Δ(DTT, Fe, S, L-Cys)	280±114
DTT, L-Tyr, GTP, PLP, SAM	Δ(DTH,Fe, S, L-Cys)	0
L-Tyr, GTP, PLP, SAM	Δ(DTT, DTH, Fe, S, L-Cys)	0
DTT, DTH, Fe, S, L-Cys, GTP, PLP, SAM	ΔL-Tyr	260±78
DTT, DTH, Fe, S, L-Cys, GTP, PLP, SAM, **DL-m-Tyr**	ΔL-Tyr	260±70
DTT, DTH, Fe, S, L-Cys, GTP, PLP, SAM, **D-Tyr**	ΔL-Tyr	672±51
DTT, DTH, Fe, S, L-Cys, GTP, PLP, SAM, **L-DOPA**	ΔL-Tyr	675±63
DTT, DTH, Fe, S, L-Cys, L-Tyr, PLP, SAM	ΔGTP	3±1
DTT, DTH, Fe, S, L-Cys, L-Tyr, PLP, SAM, **ATP**	ΔGTP	635±65
DTT, DTH, Fe, S, L-Cys, L-Tyr, GTP, SAM	ΔPLP	57±7
DTT, DTH, Fe, S, L-Cys, L-Tyr, GTP, PLP	ΔSAM	0
DTT, DTH, Fe, S, L-Cys, L-Tyr, GTP, PLP, SAM	ΔCpI apoprotein	0

Reaction mixtures contained a desalted Δ*iscR* lysate (4 mg total protein·mL^–1^), 5 µM HydE, 5 µM HydF, 50 µM HydG, 2 µM CpI apoprotein, and the standard set of extrinsic substrates except those indicated (Δ). Substrate concentrations are provided in the *Experimental Methods*. Relevant analogs were added for selected conditions and are indicated in bold font type under *Small Molecules Added*. Abbreviations: Fe, *Fe^2+^*; S, *S^2–^*; Cys, *cysteine*; Tyr, *tyrosine*; DOPA, *3,4-dihydroxyphenylalanine*.

† CpI specific activities were measured after 24 hr of incubation and are shown in units of µmol H_2_ consumed·min^–1^ mg CpI^–1^ (n = 3–6).

The positive effect of free tyrosine on *in vitro* maturation was expected since this amino acid is required for HydG-catalyzed radical SAM chemistry [6], [8], [13] and the synthesis of the CO and CN^–^ ligands coordinated to the [2Fe]_H_ subcluster [9]. Each Hyd protein was both expressed and purified separately, which precludes the possibility that the three maturases concertedly functioned *in vivo* to assemble a HydF-bound iron cluster containing the CO and CN^–^ adducts prior to the cell-free reactions [12], [15]. Thus, partial activation of the CpI hydrogenase without exogenous tyrosine indicates that the CO and CN^–^ ligands were provided by components in the ΔL-Tyr reactions, most likely associated with HydG since this is the only maturase for which tyrosine is an identified substrate. It is also possible that adventitious tyrosine accumulated during the maturation reaction as a result of protein degradation, or that purified HydG contained adventitious tyrosine.

We propose that HydG can individually synthesize an iron complex with both CO and CN^–^ ligands via a radical SAM-mediated reaction with tyrosine. We have termed this putative iron species as HydG-co. The capability of HydG to assemble HydG-co is supported by recent evidence of CO and CN^–^ sequestration within the maturase; in these previous studies, the detectable quantity of CO (10 µM) that derived from excess tyrosine (1 mM) was substoichiometric relative to the HydG concentration (60 µM), and denaturation of HydG using perchloric acid was required before the CN^–^ synthesized from tyrosine could be detected and quantified [6], [8].

FTIR spectroscopy has historically been a useful technique to analyze metalloenzymes with iron clusters containing CO and CN^–^ adducts since these species absorb in an infrared region that is not obscured by other protein-related substituents [9], [15], [21]. IR spectroscopy of 150 µM *Strep*-tag II–HydG in the as-isolated state (i.e. the purified HydG was neither concentrated nor reconstituted) did not reveal vibrational bands indicating the presence of either CO or CN^–^. We speculate that only a small percentage of the as-isolated maturase contained HydG-co, which would result in concentrations of iron-coordinated CO and CN^–^ below the detectable limits of FTIR spectroscopy. Such low concentrations of as-isolated HydG with pre-synthesized HydG-co, however, could be sufficient for partial maturation as standard reaction mixtures contained 25-fold excess HydG relative to CpI, and approximately 30% CpI was activated in the ΔL-Tyr reactions. Interestingly, using stopped-flow FTIR spectroscopy, we recently investigated the HydG-catalyzed radical SAM reaction with SAM, tyrosine, and DTH as substrates. The formation of HydG-specific Fe–CO and Fe–CN vibrational bands was observed in real-time during the first 15 minutes of the reaction. These findings, which are the focus of a separate ongoing study, strongly support our suggestion that HydG can synthesize and harbor an iron cluster with CO and CN^–^ ligands.

Besides having CO and CN^–^ adducts, we speculate that HydG-co may contain the DTMX bridge, which would indicate that HydG-co is the [2Fe]_H_ subcluster precursor that is transferred to the hydrogenase via HydF. Regarding the assembly of the [2Fe]_H_ subunit, the mechanisms for CO and CN^–^ synthesis and their attachment to an iron cluster have not been established. Dehydroglycine or a glycyl radical have each been suggested as a reactive intermediate synthesized from tyrosine [7], and it was also proposed that tyrosine could be the source of the DTMX bridge [13]. Our results when replacing L-tyrosine with DL-m-tyrosine (no additional CpI activation) or L-DOPA (complete CpI activation) suggest that the para-hydroxyl substituent is required for synthesis of the intermediate (Table 1) [20]. Further, the chiral analog D-tyrosine effectively substituted for L-tyrosine, illustrating that specific amino acid stereochemistry is not essential for the radical-based conversion of tyrosine to the reactive intermediate. Alternatively, HydG-co could be a mononuclear or dinuclear iron complex without the DTMX bridge, and this ligand would then be synthesized following transfer of HydG-co from HydG to HydF, to complete the assembly of the [2Fe]_H_ subcluster.

Similar to the effects of tyrosine, the positive influence of exogenous SAM on CpI maturation was expected as both HydE and HydG are radical SAM proteins [10]. While tyrosine has been established as an additional substrate of HydG, however, no *in vitro* biochemical evidence exists for additional substrates used by the HydE-catalyzed radical SAM chemistry.

We also examined the effects of various chemicals commonly associated with [FeS] cluster biochemistry, since both the hydrogenase as well as all three Hyd maturases harbor [FeS] centers. When exploring permutations of Fe^2+^, S^2–^, cysteine, DTT, and DTH, we observed that DTT, DTH, or Fe^2+^ plus S^2–^ enabled significant CpI activation when cysteine was also added to the reaction mixtures. That is, the following omissions were permissive for maturation: Δ(DTT, DTH), Δ(DTH, Fe, S), and Δ(DTT, Fe, S). The enhanced maturation without exogenous Fe^2+^ and S^2–^ observed in this study is different than we previously saw with our first *in vitro* system [20], which used non-purified maturases that were co-expressed under aerobic conditions. In this work, ICP-MS analysis of the as-purified maturases indicates nearly complete [FeS] cluster incorporation for each protein: 3.1 Fe per HydE peptide (76% of that expected), 3.3 Fe per HydF peptide (83% of that expected), and 6.8 Fe per HydG peptide (85% of that expected). As we saw in another previous study, our anaerobic expression methods are highly effective for producing O_2_-sensitive proteins with intact metal clusters, thus decreasing the benefits of *in vitro* [FeS] cluster reconstitution [22]. Moreover, complete CpI maturation in the absence of extrinsic Fe^2+^ and S^2–^ demonstrates the first evidence that purified HydE, HydF, and HydG are functional without the need for *in vitro* [FeS] cluster reconstitution, since the maturases are able to assemble the [2Fe]_H_ subcluster and mature the hydrogenase in a cell-free environment. It should be noted, however, that the maturase concentrations in these studies exceeded that of the CpI apoprotein by 25-fold for HydG and 2.5-fold for HydE and HydF.

Anaerobic *in vitro* studies that involve [FeS] cluster biochemistry typically employ a reducing agent such as DTT or DTH, the latter of which is also used to provide electrons for radical SAM reactions like those occurring in our hydrogenase activation system [6], [8]. We believe that DTT and DTH may also be influencing [FeS] cluster synthesis and transfer reactions, which are known to benefit from or require strong reductants [23], [24]. Interestingly, cell-free work on the FeMo nitrogenase illustrated that, when using an oxidized cell extract (similar to the aerobically-prepared lysates used in this work), an exogenous reductant such as DTH was necessary for *in vitro* nitrogenase activation [25].

We have yet to establish how PLP specifically influences *in vitro* hydrogenase maturation. Many proteins utilizes PLP as a cofactor for a diverse set of reactions [26]. Amongst these PLP-dependent enzymes are cysteine desulfurases, which are involved in [FeS] cluster biogenesis and are also significantly stimulated by a reducing agent such as DTT [27], [28]. We therefore propose that PLP is important for the cell-free synthesis of [FeS] clusters associated with the maturases or the hydrogenase apoprotein; the necessity of an *E. coli* lysate, which presumably has cysteine desulfurases, supports this theory. However, we do not rule out that PLP may be a cofactor of one of the radical SAM maturases [29], [30], perhaps as a HydE substrate and/or as the source for the DTMX bridging ligand.

The addition of GTP was tested given the classification of HydF as a GTPase [14]. Experiments with 2 mM GTP led to partial CpI maturation (∼25% activation), while substantially higher concentrations of GTP (15 mM) led to complete maturation. Surprisingly, ATP could replace GTP (Table 1), yet the HydF maturase is not capable of ATP hydrolysis [12], [14]. It is possible that nucleoside-diphosphate kinases use the ATP to convert trace amounts of adventitious GDP in the desalted extract to GTP for its role with HydF. However, either GTP or ATP was still necessary to stimulate CpI activation in the absence of HydF, the only Hyd maturase with a known capability to hydrolyze NTPs. Taken together, these data indicate that GTP and ATP are influential for uncharacterized roles in H-cluster synthesis and hydrogenase activation. Interestingly, it has been shown that the [FeS] cluster chaperones HscA and HscB stimulate cluster transfer in an ATP-dependent reaction [31]. We thus speculate that the roles of the NTPs may be associated with the unresolved *E. coli* proteins required for CpI maturation, and this is further discussed in the following section.

### Influence of HydE, HydF, HydG, and *E. coli* Cell Lysates on *in vitro* Hydrogenase Maturation

**Figure 1:**
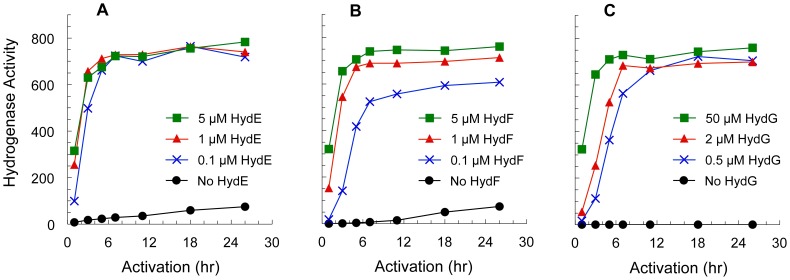
Effects of varying maturase concentrations on *in vitro* CpI activation rates. Maturation rates were examined for reactions with different maturase concentrations and 1 µM CpI. The hydrogenase specific activities (µmol H_2_ consumed·min^–1^·mg^–1^ of CpI; n = 3 reactions) were measured during a 26-hour period. Reaction mixtures contained a desalted Δ*iscR E. coli* lysate (4 mg protein mL^–1^), the standard set of extrinsic small molecular weight substrates, and Hyd maturases at the indicated concentrations. *(Fig. 1A)* Variable concentrations of HydE, 5 µM HydF, and 50 µM HydG. *(Fig. 1B)* 5 µM HydE, variable concentrations of HydF, and 50 µM HydG. *(Fig. 1C)* 5 µM HydE, 5 µM HydF, and variable concentrations of HydG.

Cell-free CpI activation was investigated when excluding each maturase (ΔHydE, ΔHydF, and ΔHydG) or a Δ*iscR E. coli* lysate devoid of the three Hyd proteins (ΔLysate). Complete activation was seen when combining all three maturases along with either a clarified lysate or a desalted lysate, while no hydrogenase was matured in the ΔLysate reactions. We also varied maturase concentrations, and nearly complete activation was seen with substoichiometric quantities of each maturase when the other two were in excess (Fig. 1). These results suggest approximately 10 turnovers for HydE and HydF as well as 2 turnovers for HydG in these experiments, indicating that each maturase can function catalytically.

The absence of hydrogenase activation in reactions devoid of an *E. coli* extract clearly indicates that the lysate is supplying components essential to the maturation process. Moreover, the complete activation of CpI that we observed when using a desalted cell extract (Table 2) suggests that the important components are likely *E. coli* proteins rather than small molecular weight substrates. However, we do not rule out the possibility that, in addition to *E. coli* proteins, adventitious small molecules that remain bound to proteins during the desalting process are also impacting cell-free hydrogenase maturation.

**Table 2:** Effects of excluding protein constituents on *in vitro* CpI activation.

Condition	Lysate	NTP	Activity†
Positive control	Clarified	GTP	740±97
Positive control	Desalted	GTP	644±141
ΔHydE	Clarified	GTP	37±17
ΔHydE	Desalted	GTP	33±22
ΔHydF	Clarified	GTP	197±53
ΔHydF	Desalted	GTP	40±16
ΔHydF	Clarified	ATP	60±2
ΔHydF	Desalted	ATP	9±1
ΔHydF	Clarified	–	0
ΔHydF	Desalted	–	0
Δ(HydE, HydF)	Clarified	GTP	0
Δ(HydE, HydF)	Desalted	GTP	0
ΔHydG	Clarified	GTP	0
ΔHydG	Desalted	GTP	0
ΔLysate	–	GTP	0

Except for the protein constituents indicated under *Condition* (Δ), reaction mixtures contained a Δ*iscR E. coli* lysate (either clarified or desalted), 5 µM HydE, 5 µM HydF, 50 µM HydG, 1 µM CpI, and the standard set of extrinsic substrates. Either no NTP or ATP (15 mM) was added instead of GTP (15 mM) for selected conditions.

† CpI specific activities are shown in units of µmol H_2_ consumed·min^–1^ mg CpI^–1^ (n = 4–8).

One explanation for the necessity of the lysate may be that [FeS] cluster machinery is required for reconstituting metal clusters associated with the maturases or the hydrogenase during the cell-free reaction. For example, ISC proteins might assemble the [4Fe–4S]_H_ subcluster in the hydrogenase H-domain, and it has been suggested that insertion of this [FeS] center precedes the insertion of the [2Fe]_H_ subcluster [17]. As previously stated, iron quantification suggests that the purified maturases appear to have fully reconstituted [FeS] clusters, although analysis of the CpI enzyme revealed that the anaerobically isolated apoprotein and re-purified holoenzyme contained 12.7±2.4 Fe per protein and 17.5±1.7 Fe per protein (88% of that expected), respectively. The addition of ∼5 Fe atoms supports the hypothesis that ISC machinery from the extract is needed to reconstitute the hydrogenase [FeS] clusters. In a separate set of experiments, we attempted to chemically reconstitute the [FeS] clusters that are presumably damaged or missing by incubating the CpI apoprotein with DTT, Fe^2+^, and S^2–^ prior to maturation. The reconstituted hydrogenase was then mixed with the maturases and the standard set of exogenous substrates. Still, no CpI was matured in the absence of the lysate. This result suggests that the cell extract impacts other reactions associated with hydrogenase maturation besides spontaneous [FeS] cluster biogenesis.

In attempts to better understand why the *E. coli* lysate is crucial for *in vitro* hydrogenase maturation, we looked at various conditions in which one or multiple Hyd maturases were excluded from the reaction mixture. Surprisingly, HydG was the only maturase always necessary for hydrogenase activation, while partial and slower activation occurred in both the ΔHydE and ΔHydF reactions (Fig. 1). Excluding both of these maturases (Δ(HydE, HydF)) led to no CpI activation (Table 2). When only HydF was excluded from the reactions (ΔHydF), we noticed that using a non-desalted (clarified) lysate led to higher CpI activities, and complete maturation could be achieved when using our previously described system [9]. That is, 100% of the CpI hydrogenase was activated in ΔHydF reactions that contained a HydG lysate, a HydE lysate, and the standard set of extrinsic substrates (data not shown). It is also striking that either GTP or ATP was still necessary when HydF – the only Hyd maturase that is known to bind and hydrolyze an NTP – was excluded from the reaction, since neither ATP nor GTP has been implicated as a substrate for HydE or HydG. Considering the necessity of both the lysate and the NTPs in the ΔHydF reactions, we speculate that one of the influential, unidentified *E. coli* proteins uses GTP or ATP as a substrate/cofactor to assist with cell-free [FeFe] hydrogenase activation.

We also investigated maturation when CpI was co-expressed in *E. coli* with (i) HydE and HydF, (ii) HydE and HydG, or (iii) HydF and HydG using established *in vivo* expression methods [22]. In each case, soluble forms of CpI and the maturases accumulated to substantial levels as determined by SDS-PAGE analysis, although no active hydrogenase was produced when any maturase was absent (data not shown). Differences in environmental conditions such as pH, maturase concentrations, and small molecule concentrations could account for the incongruence between the *in vivo* and *in vitro* maturation capability under ΔHydE and ΔHydF conditions. For example, high initial concentrations of GTP or ATP (15 mM) are important for *in vitro* hydrogenase activation, and such intracellular NTP concentrations are unlikely to exist during anaerobic growth and metabolism.

### EPR Spectroscopy of the HydF Maturase

Maturation of the CpI hydrogenase in the absence of either HydE or HydF is surprising as all three maturases are presumably essential to mature [FeFe] hydrogenases [5]. More interestingly, HydF has been considered to be the Hyd maturase that provides the [FeS] cluster framework onto which CO, CN^–^, and DTMX adducts are ligated, resulting in the formation of the [2Fe]_H_ subcluster. Considering the complexity and uniqueness of the H-cluster cofactor, and also that *E. coli* has neither a native [FeFe] hydrogenase nor the respective Hyd maturases, we believe it is unlikely that a protein from the lysate is replacing HydF to provide an [FeS] cluster scaffold for the [2Fe]_H_ subunit and also to transfer this cluster to the CpI enzyme.

The suggested role of HydF in the assembly of the [2Fe]_H_ subcluster is based upon spectroscopic studies of this maturase. Specifically, HydF was isolated following heterologous co-expression with HydE and HydG, and FTIR spectroscopy of the purified maturase revealed Fe–CO and Fe–CN vibrational modes, lending the conclusion that HydF is able to coordinate an iron center resembling the [2Fe]_H_ subcluster [12], [15]. When HydF was expressed in the absence of HydE and HydG, Broderick and coworkers used EPR spectroscopy to identify two paramagnetic species associated with photo-reduced HydF from *Clostridium acetobutylicum*. One *S* = ½ axial signal was assigned to a [4Fe–4S]^+^ cluster, and the second *S* = ½ signal was characterized as a [2Fe–2S]^+^ cluster, which the authors concluded was the [FeS] framework onto which HydE and HydG synthesize and assemble the H-cluster nonprotein ligands [12]. It is noteworthy that in that study, the *in vitro* synthesis of CO, CN^–^, or DTMX ligands onto a HydF-bound [2Fe–2S] cluster was not demonstrated. Moreover, in a separate study, Fontecave and coworkers found that DTH-reduced HydF from *Thermotoga maritima* only coordinates a single [4Fe–4S]^+^ cluster [14].

**Figure 2:**
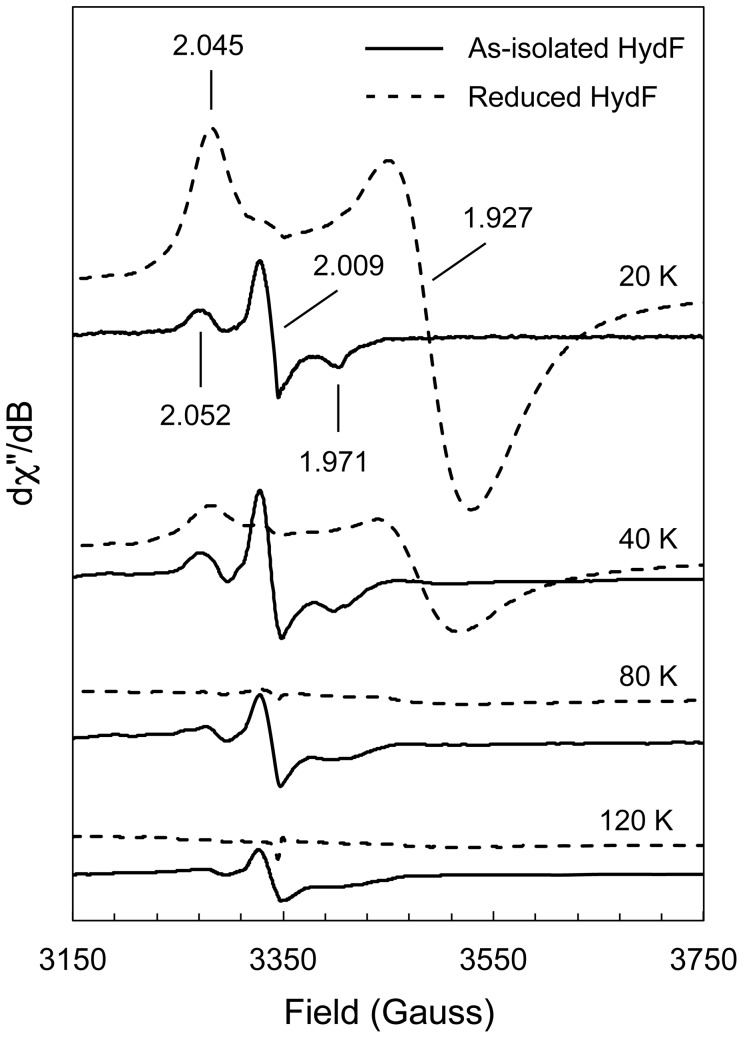
EPR spectroscopy of the *Shewanella oneidensis* HydF maturase. Continuous-wave EPR measurements were obtained at an X-band frequency (9.39 GHz). Spectra for as-isolated HydF (1.2 mM, solid lines) were measured at 20 K (50 µW), 40 K (0.5 mW), 80 K (5 mW) and 120 K (50 mW). Spectra for DTH-reduced HydF (1.1 mM, dashed lines) were measured at 20 K (200 µW), 40 K (2 mW), 80 K (50 mW) and 120 K (50 mW). The *g*-values for each signal are indicated in the 20 K spectra. Intensities were normalized to the numbers of scans, temperature, receiver gain, conversion time, modulation amplitude, and square root of microwave power. All spectra are presented without magnification of the normalized intensities. Spectra identical to those for reduced HydF were observed at 20 K and 40 K for the HydF maturase reconstituted *in vitro* with Fe^2+^, S^2–^, cysteine, PLP, and an *E. coli* lysate for 60 min prior to DTH reduction (0.80 spins per protein).

Given our findings that H-cluster synthesis and *in vitro* hydrogenase activation can occur without HydF as well as the incongruence between previous EPR studies of the maturase, we sought to spectroscopically characterize the HydF protein used in this work. Similar to the studies by the Broderick and Fontecave groups, HydF was heterologously expressed in a genetic background devoid of the HydE and HydG proteins, and therefore, the HydF maturase should not harbor a [2Fe]_H_ subcluster with CO and CN^–^ ligands. In Figure 2, EPR spectra for the as-purified HydF and the reduced HydF show different *S* = ½ species. The signal for the as-isolated HydF has *g*-values of 1.971, 2.009, and 2.052. More importantly, the line shape resembles the signal that Broderick and coworkers characterized as a [2Fe–2S]^+^ cluster and interpreted to be the iron cluster scaffold for the [2Fe]_H_ subunit. However, our as-isolated HydF sample did not contain any exogenous reducing agents, the number of *S* = ½ spins was very low (0.02 spins per protein), and the *S* = ½ signal centered at *g* = 2.009 disappeared upon reduction with DTH, which indicates that it does not represent a [2Fe–2S]^+^ center. For the DTH-reduced HydF maturase, the 20 K spectrum shows an axial *S* = ½ signal (*g* = 1.927, *g* = 2.045; 0.85 spins per protein) consistent with the [4Fe–4S]^+^ cluster identified in the previous HydF studies [12], [14]. Similar to Fontecave and coworkers and despite our attempts to reconstitute a [2Fe–2S] cluster *in vitro*, we did not observe a second signal indicative of this type of [FeS] center.

Based on the EPR spectroscopy, the Fe content results (3.3 Fe per HydF), and the *in vitro* hydrogenase maturation data for ΔHydF conditions, we propose that HydF neither coordinates a [2Fe–2S] cluster when anaerobically produced in the absence of HydE and HydG, nor does HydF supply an [FeS] cluster framework onto which the H-cluster nonprotein ligands are assembled. It is worth noting that with our cell-free system, the three maturases activated the hydrogenase without extrinsic Fe^2+^ and S^2–^ as well as with substoichiometric quantities of HydF relative to CpI, together suggesting that the [FeS] cluster framework for the [2Fe]_H_ subcluster already exists on either the purified HydE or HydG maturase. We also believe that the as-isolated HydF paramagnetic species observed in this study, which appears to be the same signal characterized by Broderick and coworkers as that for a [2Fe–2S]^+^ cluster [12], represents either a protein-associated radical or a [3Fe–4S]^+^ cluster. A similar *S* = ½ species, identified as a cysteinyldisulfide radical, was observed upon Fe(CN)_6_^3–^ oxidation of the *Azotobacter vinelandii* ferredoxin I [32]. A nearly identical spectrum was also measured for as-isolated PqqE, a radical SAM protein that harbors only [4Fe–4S] clusters, and the authors suggested that a [3Fe–4S]^+^ cluster accounts for this signal [33]. At this time, we are exploring other measures such as pulse and high-frequency EPR spectroscopy to more thoroughly characterize the *S* = ½ signal centered at *g* = 2.009 for the as-isolated HydF maturase.

### Model for the [FeFe] Hydrogenase Biosynthetic Pathway

**Figure 3:**
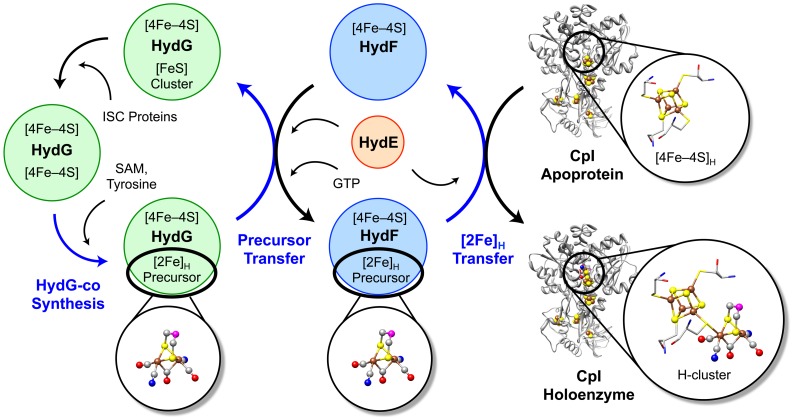
Schematic illustrating the proposed H-cluster biosynthetic pathway and roles of the Hyd maturases for [FeFe] hydrogenase activation. Blue arrows indicate the steps directly associated with the synthesis and translocation of the [2Fe]_H_ subcluster. The H-cluster CO and CN^–^ ligands, and possibly the DTMX bridge, are generated from tyrosine during HydG-catalyzed radical SAM chemistry. These adducts are assembled onto a HydG-bound [4Fe–4S] cluster to form HydG-co, a [2Fe]_H_ subcluster precursor. HydG transfers this precursor to HydF in a process during which HydF-catalyzed GTP hydrolysis may cause a conformational change that facilitates the interaction of HydF with HydG and/or HydE in order to acquire HydG-co. HydF then inserts the [2Fe]_H_ precursor into the hydrogenase apoprotein, resulting in the maturation of active [FeFe] hydrogenase. We hypothesize that by utilizing electrons derived from radical SAM chemistry, HydE assists with [2Fe]_H_ precursor transfer amongst HydG, HydF, and the hydrogenase apoprotein. Following the transfer of HydG-co to HydF, the HydG CTD [4Fe–4S] cluster scaffold is likely reconstituted by the host cell ISC machinery. Ball-and-stick representations of the H-cluster and its precursors are depicted with the following color scheme: Fe, brown; S, yellow; C, gray; O, red; N, blue; unknown atom (X), magenta.

The surprising observations that HydE and HydF – purportedly essential proteins for producing active [FeFe] hydrogenases – are not required for *in vitro* maturation lends us to conclude that these two maturases neither synthesize the CO, CN^–^, and DTMX ligands nor provide the iron cluster scaffold for the [2Fe]_H_ precursor. Rather, as illustrated in the Figure 3 schematic, we propose that HydG can independently assemble an iron compound precursor with both CO and CN^–^ adducts (i.e. HydG-co). The HydG maturase has two [FeS] clusters: an N-terminal domain (NTD) [4Fe–4S] radical SAM cluster and a C-terminal domain (CTD) cluster that is also presumably a [4Fe–4S] center [34], which has been established to be necessary for the generation of CO from tyrosine [7]. We believe that the CTD [FeS] cluster is likely the scaffold for HydG-co. However, we have yet to establish the precise structure of the ligand-coordinated [2Fe]_H_ precursor. If HydG-co is a [2Fe] cluster that derives from the CTD [4Fe–4S] center, we believe that the presence of a dithiol bridge would be required before CO and CN^–^ would coordinate to form the [2Fe]_H_ subunit, which is perhaps synthesized from tyrosine by the previously suggested mechanism [13]. HydG-co may also be a mononuclear iron complex with only the CO and CN^–^ ligands. As previously stated, the characterization of HydG-co is ongoing work and the subject of a separate study.

We propose that HydF functions as a transferase to stabilize HydG-co and catalyze its translocation from HydG to the hydrogenase apoprotein. This hypothesis is based on (i) our observation that CpI is matured in the ΔHydF reactions, (ii) our EPR spectroscopic evidence that HydF expressed in the absence of HydE and HydG does not coordinate a [2Fe–2S] cluster, and (iii) our suggested roles for the HydG maturase. HydG may also be able to transfer HydG-co directly to the hydrogenase, which could explain the CpI activation in reactions without HydF. Although GTP does not affect transfer of the [2Fe]_H_ precursor from HydF to the hydrogenase apoprotein [12], it is possible that GTP hydrolysis influences the interaction of HydF with HydG and/or HydE in order to acquire HydG-co.

It was recently proposed that HydE synthesizes the DTMX ligand by bridging the sulfides of a HydF-bound [2Fe–2S] cluster [11], [12]. No present theories relating to this function of HydE, however, are based on experimental evidence, and at this time, we do not have experimental data that directly support any suggested maturation role for HydE. It is possible that HydG-co is not the complete [2Fe]_H_ subunit, and upon translocation to HydF, both the HydE and HydF maturases work together to assemble the DTMX bridge. Yet, given our result that CpI maturation can occur in the absence of HydE, it seems likely that another protein creates the DTMX bridging ligand. We therefore postulate that HydE assists with translocation of the [2Fe]_H_ precursors amongst HydG, HydF, and the hydrogenase apoprotein (Fig. 3), and we further speculate that SAM is the only substrate of HydE.

### Conclusions

In this work, we describe the first use of separately produced and purified Hyd maturases for combined cell-free H-cluster synthesis and [FeFe] hydrogenase maturation. By providing evidence for maturase turnover, we establish the validity of this model system, which enables us to reliably characterize how different components influence hydrogenase activation. Although we have yet to identify the required *E. coli* proteins and determine how essential small molecules affect maturation (e.g. GTP and PLP), we are currently investigating these unknowns in order to reconstruct the reaction sequence with a defined set of proteins and substrates. Nevertheless, our studies have rendered several key insights. We illustrate that *in vitro* hydrogenase maturation can proceed without HydE, HydF, or exogenous tyrosine. From these findings, we hypothesize a new model for [FeFe] hydrogenase activation, in which HydG assembles an iron complex with CO and CN^–^ ligands that is a precursor to the H-cluster [2Fe] subunit. Future studies can also utilize this cell-free system in combination with biochemical analyses such as HPLC and LC-MS to focus on characterizing maturase interactions and elucidating the complete biosynthetic reaction sequence of the H-cluster cofactor.

## Experimental Methods

### Production of *E. coli* Cell Lysates and Purified Proteins

Cell cultures were performed as 4 L batch fermentations using a 5 L BioFlo 3000 fermentor (New Brunswick Scientific). Growth medium consisted of LB Miller with 50 mM MOPS/KOH buffer (pH 7.4), 25 mM glucose, 500 mg·L^–1^ ferric ammonium citrate, and the appropriate antibiotics [9], [22]. Each Hyd maturase as well as the CpI apoprotein were produced separately using *E. coli* strain BL21(DE3) Δ*iscR::kan* and subsequently purified as previously described [9].

*E. coli* strain BL21(DE3) Δ*iscR::kan* and the growth medium described above were also used to produce lysates that did not contain the heterologous maturases, and all steps were done under aerobic conditions. Cells were grown at 25°C until an OD_600_ of ∼2, harvested, pelleted, and lysed using BugBuster® Master Mix (Novagen) supplemented with 50 mM Hepes buffer (pH 7.6). Clarified lysates were produced by centrifuging crude lysates at 30,000×g for 30 min. Desalted lysates were made by buffer exchanging the clarified lysate twice with PD-10 desalting columns (GE Healthcare) and subsequently concentrating the desalted lysate using a stirred cell and a 5 kDa MWCO membrane (Amicon).

Previously described pET-21(b) *hydA–Strep*-tag II, pET-21(b) *hydE–Strep*-tag II, pET-21(b) *hydF–Strep*-tag II, and pET-21(b) *Strep-*tag II–*hydG* constructs were used for the anaerobic production of the *Clostridium pasteurianum* HydA (CpI) apoprotein and the *Shewanella oneidensis* HydE, HydF, and HydG maturases [9]. CpI–*Strep*-tag II apoprotein was anaerobically isolated and concentrated to 2 mg·mL^–1^ (30 µM) using a stirred cell and a 30 kDa MWCO membrane. Concentrated CpI was anaerobically sealed, flash frozen using liquid nitrogen, and stored at –80°C. The anaerobically purified maturases were not concentrated since protein precipitation was occasionally observed during this step. Instead, the maturases were anaerobically sealed and flash frozen at their as-purified concentrations: 2 mg·mL^–1^ HydE–*Strep-*tag II (40 µM), 2 mg·mL^–1^ HydF–*Strep-*tag II (40 µM), and 12 mg·mL^–1^ *Strep-*tag II–HydG (150 µM). Protein concentrations were estimated using the Bradford assay [35] along with a bovine serum albumin standard. Iron quantification for the hydrogenase and the maturases was done using inductively-coupled plasma mass spectrometry (ICP-MS) at the UC Davis Interdisciplinary Center for ICP-MS. In order to quantify the iron content of active CpI, the hydrogenase was matured and re-purified using our analogous *in vitro* system that utilizes non-purified maturases without *Strep*-Tactin purification tags [9].

### Cell-free H-cluster Synthesis and [FeFe] Hydrogenase Maturation

*In vitro* activation of the CpI apohydrogenase was carried out at 26°C in an anaerobic glove box (Coy Laboratory Products), and reaction volumes were 50–100 µL [9]. Standard reaction conditions consisted of proteins and substrates at the following concentrations: 5 µM HydE–*Strep-*tag II, 5 µM HydF–*Strep-*tag II, 50 µM *Strep-*tag II–HydG, 20% vol·vol^–1^ lysate (4 mg total protein·mL^–1^) from *E. coli* strain BL21(DE3) Δ*iscR::kan*, 1 mM Fe^2+^, 1 mM S^2–^, 1 mM DTT, 2 mM DTH, 2 mM SAM, 2 mM L-cysteine, 2 mM L-tyrosine, 15 mM GTP, 2 mM PLP, and 1–2 µM CpI apoprotein. CpI activities were quantified using a methyl viologen-based H_2_ uptake assay, and data are presented as hydrogenase specific activities. We previously determined that a specific activity of ∼675 µmol H_2_ consumed·min^–1^·mg^–1^ of CpI indicates complete hydrogenase maturation [9].

### *In vivo* Co-expression of Selected Maturases with the [FeFe] Hydrogenase

CpI was co-expressed with two or three Hyd maturases using previously described methods for high-yield hydrogenase expression in the *E. coli* strain BL21(DE3) Δ*iscR::kan* [22]. Following cell lysis, CpI activities were measured with the H_2_ uptake assay. The maturase expression constructs for these experiments were pACYCDuet-1–*hydGx*–*hydF*, pACYCDuet-1–(empty cloning site)–*hydEF*, pACYCDuet-1–*HydGx*–*hydE*, and pACYCDuet-1–*hydGx*–*hydEF*.

### EPR Spectroscopy

EPR spectroscopy was done at the CalEPR Center in the Department of Chemistry at the University of California, Davis. Continuous-wave spectra were collected at an X-band frequency (9.39 GHz) with a Bruker Biospin Elexsys E500 spectrometer equipped with a cylindrical TE_011_-mode resonator (SHQE-W), an ESR-900 liquid helium cryostat, and an Oxford Instruments ITC-5 temperature controller. Measurement parameters were typically as follows: temperatures, 10–120 K; microwave powers, 10 µW–100 mW; modulation amplitudes, 0.2–0.5 mT; sweep widths, 300–400 mT; conversion time, 80 ms; sweep time, 82 s. Spin quantitation was based on Cu-EDTA standards measured at 80 K and 25 µW microwave power. HydF samples for EPR spectroscopy were prepared using the described expression and purification procedures. Without freezing and thawing the protein after purification, the as-isolated HydF was concentrated to 1.2 mM using 500 µL centrifugal ultrafiltration units (Amicon) with a 30 kDa MWCO membrane.
